# An observational cohort study of factors associated with patients’ reports of being offered lumbar spine surgery during orthopaedic spine consultations for chronic low back pain

**DOI:** 10.1177/20494637261472434

**Published:** 2026-08-03

**Authors:** Kathrin Braeuninger-Weimer, Naffis Anjarwalla, Asef Al-Ani, Douglas Evans, Hanna Rooslien, Tamar Pincus

**Affiliations:** 1 Department of Psychology, 3162 Royal Holloway, University of London, Egham, Surrey, UK; 2 Department of Orthopaedics Wexham Park Hospital, Frimley Health NHS Foundation Trust, Slough, Berkshire, UK; 3 School of Psychology, 7423 University of Southampton, Southampton, UK

**Keywords:** chronic primary low back pain, surgical decision-making, spine surgery consultation, clinical evaluation in spine surgery, patient selection for spine surgery, non-specific low back pain, pre-operative assessment in spine surgery

## Abstract

**Background:**

Guidelines do not recommend lumbar spine surgery for people with chronic low back pain (CLBP). It remains unknown how these guidelines are implemented, and which factors affect the decision to offer surgery.

**Objectives:**

This study examined factors associated with patients reporting that lumbar spine surgery was recommended during specialist consultation for CLBP.

**Methods:**

Clinical, demographic and psychosocial factors were measured pre-consultation in people referred with CLBP for their first consultation with orthopaedic spine clinicians in secondary care in eight hospitals. Patients reporting leg pain, previous spinal surgery, or other specific spinal pathology were excluded. Data were analysed using binomial logistic regression. In addition, three surgeons independently rated a subsample of the MRI reports as suitable or unsuitable for lumbar spine surgery, blinded to clinical background of patients and to the consultation outcome.

**Results:**

A total of 424 patients were included in the analysis, of whom 96 reported being offered lumbar spine surgery. Patients were more likely to report being offered surgery if they were older, had a longer pain duration, had consulted more widely, and had stronger positive expectations of surgery. Pain intensity, disability, depression, and anxiety were not associated with the decision to offer surgery. The model correctly classified 80.6% of cases. In the subsample analysis based on MRI reports alone, the proportion judged as potentially suitable for lumbar spine surgery (20%) was similar to the actual consultations (22%), but concordance on who was offered surgery was poor (27%).

**Conclusion:**

In this cohort of people with CLBP without leg pain, patient perception that surgery was offered during the consultation was associated with a longer history of pain and a greater number of prior consultations. Surgical offer was patient-reported and surgeons’ rationale was not captured. Subsample findings suggest that MRI findings alone should not drive surgical decision-making in CLBP.

## Introduction

Chronic low back pain (CLBP) remains the leading cause of years lived with disability worldwide^1^ and is among the most common reasons for seeking healthcare.^2^ Recommended treatment emphasises reassurance, education, lifestyle modifications, and increased physical activity, while discouraging overuse of imaging, opioids, and surgery.^3^ Who will benefit from lumbar spine surgery remains unknown: research indicates that neither the duration of pre-operative conservative treatment nor pre-surgical pain intensity is associated with post-operative outcomes in spinal surgery patients.^4^ This raises questions about the criteria used to determine surgical candidacy in people with CLBP. In some cases, non-radicular spinal conditions (e.g. stenosis) may still warrant surgical assessment.

In the UK, guidelines recommend against offering spinal fusion and do not support clinicians routinely offering imaging in people with CLBP, without red flags or radicular symptoms, unless the results are expected to change management.^5^ A recent series of papers in the Lancet described imaging and surgery for CLBP as costly and harmful, based on the evidence that most CLBP is unrelated to specific identifiable spinal abnormalities.^3,6^ In clinical practice, MRI findings frequently indicate nerve involvement in individuals without leg pain or radicular symptoms, underscoring the limited specificity of imaging for clinical decision-making. A recent, large population-based cohort study examining the relationship between MRI findings and LBP concluded that, over time, most MRI findings were not associated with future LBP severity, regardless of the presence or absence of baseline pain,^7^ suggesting limited prognostic value of these findings when imaging is obtained routinely. Participants with CLBP in this study may have a primary pain syndrome or a structural cause, without radiculopathy or red flags.

A recent international survey of spine surgeons suggested that 36% prioritise ordering an MRI as the first step for people with CLBP without red flags, surpassing those who opted for conservative treatments.^8^ A study of 405,965 people with CLBP indicated that early MRI use was associated with excess surgery, greater opioid use, worse outcomes, and higher healthcare costs.^9^ These findings raise concerns about the extent to which MRI findings, rather than clinical symptoms and function, influence clinical decision-making across specialist settings. As consultations for CLBP occur not only in surgical clinics but also in chronic pain and musculoskeletal services, understanding how imaging and patient expectations influence clinical decision-making across these contexts is essential.

Evidence from a somewhat dated large cohort study of 4987 patients with CLBP suggests that female sex assigned at birth, previous back surgery, high-intensity leg pain, somatisation, and positive treatment expectations significantly increase the likelihood of being offered spinal surgery.^10^ Psychological factors, such as anxiety and depression, are associated with lumbar spine surgery outcomes (excluding sciatica), ^11^yet standardised psychosocial assessments remain underutilised in surgical decision-making.^12,13^ Many orthopaedic surgeons lack confidence, training, and communication strategies to assess and discuss psychosocial factors, even when standardised tools are available.^14,15^ Evidence also suggests that orthopaedic surgeons often find it challenging to address psychological factors and to communicate the decision to decline surgery for people with CLBP.^16^

This study investigates:

What proportion of people with CLBP without radicular pain referred to orthopaedic teams report being offered lumbar spine surgery?

Which patients’ characteristics are associated with patients’ reports of being offered lumbar spine surgery?

To what extent do surgeons’ recommendations based on imaging findings interpreted in isolation agree with patients’ reports of being offered lumbar spine surgery following consultation?

## Methods

### Study design

This study was part of a multisite prospective observational cohort study, published elsewhere,^17^ including patients consulting secondary-care orthopaedic spinal clinicians for their CLBP for the first time.

### Setting

Patients were recruited from 8 different NHS hospitals between June 2017 and June 2019. The hospitals were distributed across 3 NHS Foundation Trusts (2 Trusts each included 3 hospitals and 1 Trust included 2 hospitals) and were located across semi-urban and rural areas, reflecting the diverse catchment populations of South-West England. All hospitals are expected to follow national guidelines, including NICE recommendations. While orthopaedic teams may overlap within the same Trust, there was no overlap between the different Trusts, therefore hospitals within each Trust may have used their own decision-making processes.

**Figure 1.**
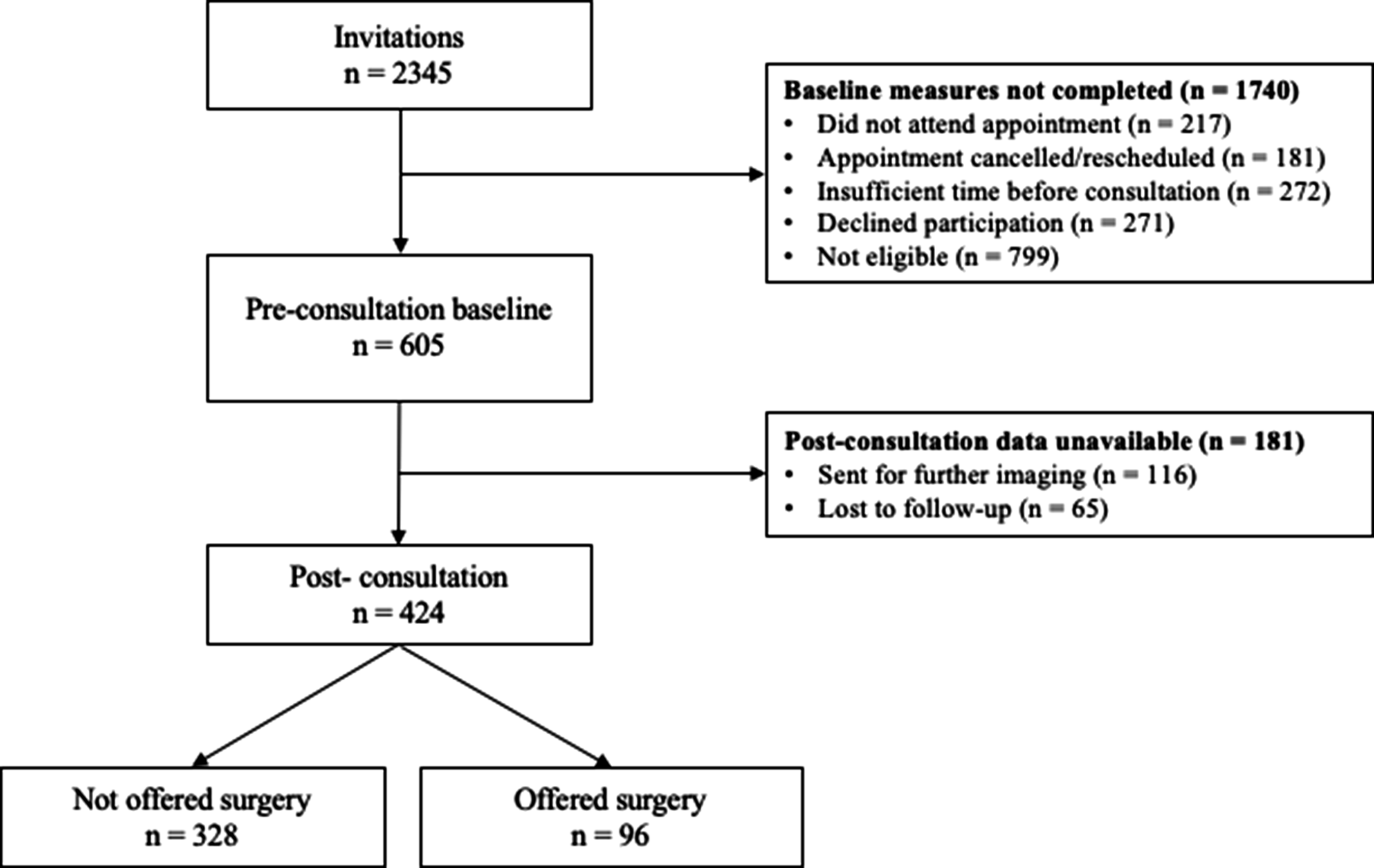
Flow of patient data throughout the study. Note: Patients sent for further imaging (*n* = 116) or lost to follow-up (*n* = 65) were excluded from the analysis due to missing post-consultation data.

Participating hospitals employed three orthopaedic teams specialising in spine care, including 13 consultant orthopaedic surgeons (11 men and 2 women) and nine advanced physiotherapy practitioners (APP) (7 women and 2 men). All clinicians subspecialised in spinal care. All patients consulting outpatient orthopaedic spinal settings were sent letters of invitation. Patients interested in participating provided written consent and completed the baseline questionnaire pre-consultation. Participants were followed-up post-consultation within 1 week. The primary outcome was patients’ reports of being offered lumbar spine surgery (see Figure 1 for participant flow chart). The study was adopted to the National Institute for Health Research (NIHR) portfolio and data collection was assisted by nurses from the Comprehensive Local Research Network (CLRN). Ethical approval for this study was granted by NHS Bromley Research Ethics Committee (16/LO/1833) and by the ethics committee at Royal Holloway, University of London. The study was conducted by a multidisciplinary authorship team, including practising orthopaedic spine surgeons, health psychologists, and a data scientist.

### Participants

The ICD-11 classification defines chronic primary low back pain (CPLBP, MG30.02) as LBP persisting for more than 3 months without an identifiable structural cause and often associated with functional disability or emotional distress.^18^ All patients included in the study had LBP as the main and only reason for consulting with the spinal orthopaedic team. Patients were excluded prior to baseline data collection, if they reported leg pain, other injuries, or any complaint other than LBP as their main reason for consulting the orthopaedic team. In addition, patients who reported pregnancy, cauda equina syndrome, ankylosing spondylitis, other inflammatory spinal conditions, previous spinal surgery, end-of-life disorders, cognitive impairment, or those who were under 18 years old, or unable to speak or read English, were excluded from the study. Patients who reported post-consultation that surgical decision was pending due to referral for further imaging were excluded from the study due to logistical challenges associated with long NHS imaging wait times. Finally, during the consultation, five patients were excluded based on imaging findings that, according to the surgeon, suggested serious spinal pathology, such as malignancy, fractures, infection, or cauda equina syndrome, and were explicitly deemed not to have CLBP.

### Variables

At baseline before the consultation, patients provided demographic information including age, sex assigned at birth, and education status. They also indicated the duration of their current episode and the number of previous consultations with different professionals about low back pain. They were asked to provide scores on a single-item measure ‘to what extent do you expect surgery from the consultation you are about to attend’ (numeric 7-point scale, 1 = ‘not at all’, 7 = ‘a great deal’). For analysis, this was treated as a continuous variable, as assumptions of linearity were not violated. In addition, they reported their average weekly pain intensity level (numeric 11-point scale,^19^ disability (Roland-Morris Disability Questionnaire [RMDQ]),^20^ and distress (Hospital Anxiety and Depression Scale [HADS]).^21^ For the purpose of analyses, the HADS scores were treated as two separate measures, anxiety and depression, based on previous research.^22^ The HADS is a 14-item self-report questionnaire designed to evaluate the severity of anxiety and depression in people with physical health problems in the past week (7 items relating to anxiety; 7 items relating to depression). Respondents rate their answers on a 4-point Likert scale with item scores ranging from 0 to 3. The sub-scale scores range from 0 to 21, whereby higher scores indicate greater levels of distress. The RMDQ is a widely used disability scale for low back pain.^23^ The measure contains 24 items measuring activity limitations associated with back pain, for which respondents tick those that apply to them that day. Each item is dichotomously scored (yes/no) and the total score is calculated by the total number of items checked (minimum of 0 meaning no disability up to a maximum of 24).

### Data sources and measurements

To reduce potential sources of bias, all measures were collected immediately before and within one week after patients’ first secondary-care consultation with orthopaedic spine clinicians.

At 1-week post-consultation, patients were presented with tick-box items reporting the outcome of their consultation with orthopaedic spine clinicians, including the item ‘I was recommended to undergo surgery (yes/no)’.

In line with standard care in the UK, all MRI scans received a formal radiologist report. Imaging on its own is not used to determine surgical candidacy. In this component of the study, imaging-based assessment was used to isolate and examine variability in the interpretation and classification of MRI radiology reports, rather than to reflect real-world surgical decision-making. Three orthopaedic surgeons in spine speciality training (registrars) (including AA, DE), supervised by a senior spine surgeon (NA) in the interpretation of the imaging reports, were presented with a sample of anonymised radiology reports from the MRI scans. Imaging reports (*n* = 134) were reviewed across the three registrars, with distribution based on feasibility and final selection number determined pragmatically by registrar availability and data completeness. The sample included imaging reports from six of the eight participating hospitals. Registrars had no involvement in the clinical consultations and were blinded to the patients’ clinical history and the consultation outcome in reference to treatment. Surgical decisions were made during the consultation by consultant surgeons, thus agreement patterns may differ if the imaging exercise were conducted by consultants.

They were asked whether they would offer lumbar spine surgery based on the imaging reports alone and to classify each report into three categories based on imaging findings, rather than to identify clinical red flags:

a. Serious spinal pathology including tumour, infection, or fracture,
b. Back pain with nerve pain affecting the leg,
c. Low back pain (precise cause cannot be identified, without red flags and radicular symptoms).

## Statistical methods

All analyses were performed using SPSS version 23.^24^ Missing values were excluded from the analyses. Baseline patient characteristics were compared between responders and non-responders at the 1-week post-consultation follow-up using logistic regression to assess differences between those who completed the follow-up measures and those who did not. The minimum required sample size was estimated using the commonly applied ‘20 events per variable’ rule of thumb for logistic regression.^25^ Preliminary assumption checking for the binomial logistic regressions involved: (1) using the Box-Tidwell (1962) approach to assess linearity between the outcome variable and the logit transformation of the predictor variables, (2) assessing the correlation coefficients (>0.7) and Variance Inflation Factor (VIF) (<5) values, to ensure there was no multicollinearity, (3) examining the ‘case wise diagnostic table’ for standardised residuals (±2 SD).^26^ Model fit was evaluated using the Hosmer-Lemeshow test and Nagelkerke R^2^ to assess the goodness-of-fit of the regression model.

A hierarchical multivariable logistic regression, on a dichotomous outcome variable (surgery vs. no surgery), was performed to ascertain the effects of 10 predictor variables, including age, sex assigned at birth, education, duration, number of previous consultations for low back pain, surgical expectations, pain, disability, anxiety, and depression.

## Results

Patients who did not complete the 1-week post-consultation follow-up measures (*n* = 65, 10.7%) were excluded from the analyses. The final analysis included data from 424 patients, of whom 96 (22.6%) reported being recommended to undergo lumbar spine surgery and 328 (77.4%) reported that surgery was not recommended (see Table 1 for baseline and clinical characteristics). These classifications were based on patients’ reports and may not always reflect the surgeon’s intended recommendation. Of those who did not report being offered surgery, 133 (40.5%) reported discharge from care and 195 (59.5%) reported referral for other treatments, such as physiotherapy or injections. Missing values for each variable are presented in Table 1.

**Table 1.** Demographics and clinical profiles.

​	Offered surgery group (*n* = 96)	No offered surgery group (*n* = 328)	Total (*n* = 424)
Age			
Mean (SD)	57.4 (16.7)	54.6 (17.2)	55.21 (17.14)
Range	21–90	19–88	19–90
Missing (%)	2 (2.1)	3 (0.9)	5 (1.2)
Sex assigned at birth *n* (%)			
Female	56 (58.3)	190 (57.9)	245 (58)
Males	40 (41.7)	138 (42.1)	178 (42)
Missing (%)	0	0	0
Education *n* (%)			
Left school before 16	38 (39.6)	121 (36.9)	159 (37.5)
A-level equivalent	23 (24.0)	59 (18.0)	82 (19.3)
Higher education	32 (33.3)	124 (37.8)	156 (36.8)
Missing (%)	3 (3.1)	24 (7.3)	27 (6.4)
Duration of current episode of back pain *n* (%)			
Less than 3 months	8 (8.3)	57 (17.4)	65 (15.3)
More than 3 months	85 (88.5)	267 (81.4)	352 (83.0)
Missing (%)	3 (3.1)	4 (1.2)	7 (1.7)
Number of pre- treatments			
Mean (SD)	2.5 (1.6)	2.1 (1.5)	2.2 (1.6)
Missing (%)	2 (2.1)	2 (0.6)	4 (0.9)
Pain			
Mean (SD)	6.8 (2.0)	5.7 (2.5)	5.92 (2.47)
Missing (%)	2 (2.1)	2 (0.6)	4 (0.9)
Disability (RMDQ, 0–24)			
Mean (SD)	13.4 (5.4)	10.3 (6.1)	11.02 (6.07)
Missing (%)	2 (2.1)	2 (0.6)	4 (0.9)
Anxiety (HADS-A, 0–21),			
Mean (SD)	8.4 (4.3)	7.5 (4.7)	7.67 (4.66)
Missing (%)	3 (3.1)	5 (1.5)	8 (1.9)
Depression (HADS-A, 0–21)			
Mean (SD)	7.5 (3.8)	6.2 (4.0)	6.5 (4.00)
Missing (%)	3 (3.1)	5 (1.5)	8 (1.9)

A binary logistic regression was utilised to see whether patient history or characteristics could predict those who provided follow-up data versus those who did not. The model was not significant, χ^2^ (8) = 6.276, *p* = .616. Patients’ characteristics and history did not predict responders from non-responders. Patients who reported being referred for further MRI imaging (*n* = 116, 19.2%) were also excluded from the analyses. A binary logistic regression was used to see whether patient history or characteristics could predict those who were provided follow-up data from those who did not. The model was not significant, χ^2^ (8) = 12.186, *p* = .143. Patients’ characteristics and history did not predict whether patients reported being referred for further imaging or not.

The assumption of linearity in the logit, assessed using the Box-Tidwell test, was not violated (*p* < .05). The Hosmer and Lemeshow Test was not significant (*p* = .616), which meant the model is a good fit. The logistic regression model was statistically significant, χ^2^ (12) = 76.840, *p* < .001, with a Nagelkerke R^2^ of 28.6%, indicating model fit. Participants who reported being offered surgery were older (p = .012), had pain for longer (*p* = .037), had consulted more clinicians (*p* = .032), and had more positively expected surgery (*p* = .001). The model correctly classified 80.6% of the cases overall. Sensitivity (surgery cases correctly classified) was 36.5% and the specificity (not offered surgery cases correctly classified) was 94.0% (see Table 2 for Beta, standard error, Wald, and 95% confidence intervals for odds ratios). The low sensitivity likely reflects the small proportion of patients reporting being offered surgery and highlights the model’s descriptive rather than predictive utility. Patients who reported being offered surgery had higher scores on the expectations of surgery measure (M = 4.6, SD = 2.2) compared to those who reported not being offered surgery (M = 2.9, SD = 1.9). Expectations of surgery were not significantly associated with age, sex assigned at birth, or pain duration, and showed only a modest correlation with pain intensity (r = .35).

**Table 2.** Binominal regression model predicting patients who were recommended surgery. Notes: *n* = 424; B = unstandardised regression coefficient; 95% CI = confidence interval. Offered surgery = 1, Not offered surgery = 0. **p **p < .01, *p < .05.

Variables	*B*	*SE*_*B*_	*Wald*	*95% CI*
Age	.023*	.009	6.302	[1.005, 1.041]
Sex assigned at birth	.026	.297	.007	[.573, 1.837]
Education	.418	.301	1.925	[.842, 2.739]
Duration	.742*	.356	4.334	[1.044, 4.222]
Number of previous consultations	.890*	.416	4.587	[1.078, 5.499]
Extent of surgery expected	.438**	.076	33.460	[1.336, 1.798]
Pain level	.031	.077	.165	[.887, 1.201]
Disability	.027	.033	.673	[.963, 1.097]
Anxiety	−.004	.047	.009	[.908, 1.092]
Depression	.023	.054	.175	[.920, 1.138]

We were able to obtain a random sample of 134 MRI reports from the entire cohort, including patients reporting being offered lumbar spine surgery (*n* = 30) and those reporting discharged or referred for other treatment (*n* = 104). For the re-classification using 134 MRI reports alone, out of the 30 cases in which surgery was reported to have been offered, only 8 were offered surgery based on MRI reports alone (27.0% concordance). In addition, 19 (18.3%) scans were considered surgical candidates, whereas the patient-reported consultation outcome indicated that surgery was not offered (see Table 3). The majority of patients were coded as having nerve pain affecting the leg (74%), and the remaining were considered to have back pain for unknown reasons (23.0%). Three MRI reports were classified into the serious spine pathology category by the reviewing registrars.

**Table 3.** Surgeons’ independent assessment of MRI reports in isolation compared with patient-reported surgical offer following consultation.

Patient-reported surgical offer		Surgeons’ assessment of whether scan indicates need for surgery	
		Yes	No
Offered surgery	30	8 (26.7%)	22 (73.3)
Not offered surgery	104	19 (18.3%)	85 (81.7%)

## Discussion

The findings suggest that people who were older, had a longer current pain duration, consulted more extensively, and reported stronger expectations that surgery would be recommended were more likely to report being offered lumbar spine surgery in the UK. The strongest indicator of patients’ reports of being offered lumbar spine surgery appears to be the extent to which patients expected it, in combination with longer pain duration and a higher number of previous consultations. This appears to be independent of pain intensity and functional disability, which were not associated with the decision to offer lumbar spine surgery. The findings also suggest that patients’ psychological factors, such as depression and anxiety, do not influence patients’ reports of being offered surgery. Finally, in this study setting, imaging was carried out for all patients and lumbar spine surgery was offered to one in five patients despite the exclusion of those with reported leg pain.

The findings suggest that patients’ reports of being offered lumbar spine surgery were associated with longer pain duration and higher healthcare utilisation previously. Patients with a longer history of chronic pain may have higher expectations of being offered surgery and their preference could have been conveyed to the surgeons during the consultation. Patients’ expectations may influence the consultation dynamic without necessarily reflecting a direct request for surgery. The relationship between expecting surgery (from patients) and offering lumbar spine surgery requires further research as part of exploring how shared decision-making can be implemented in surgical settings. A systematic review of over 13,000 consultations found that less than a third of patients considered the decision-making to be shared.^27^ It may also be an indication of lumbar spine surgery being offered as a last resort, especially when combined with patients wanting to undergo surgery. Despite this, the evidence from over 3000 patients in Europe suggests no association between pain duration and the number of previous treatments with positive surgery outcomes.^4^ There may be, therefore, a disconnect between surgeons’ considerations of who is most suitable for lumbar spine surgery, and the evidence regarding who benefits from it. However, it is difficult to establish the exact link between these factors and the decision-making process without consulting clinicians directly.

The results indicated that factors such as participants’ distress levels were not associated with patients’ reports of being recommended lumbar spine surgery. However, evidence suggests that it is important for these factors to be considered and formally assessed: a systematic review and meta-analysis of 29 studies revealed that anxiety and pain catastrophizing were associated with the transition to chronic post-operative pain.^28^ The observed effects were strongest in musculoskeletal surgery. This is also in line with evidence suggesting spine surgeons rarely use pre-operative psychological assessments for patients,^29^ struggle to accurately assess psychological distress even when using standardised tools,^12,30^ and often underestimate the effects of distress on pain outcomes.^16^ A longitudinal cohort study on the predictive role of executive functions and psychological factors in CLBP after surgery found that pain catastrophizing, state anxiety, healthcare-related fears, and visual attention significantly predicted post-surgical pain intensity and its sensory, affective, and disability components.^31^ A recent systematic review examining the prognostic value of psychosocial factors on the change in pain and/or disability in people with CLBP concluded that fear of movement, self-efficacy, catastrophizing and depression were consistently reported to predict disability outcomes irrespective of the type of intervention.^32^ These findings highlight the importance of addressing pre-surgical psychological factors, considering their association with pain and disability regardless of type of intervention.

In this study, approximately 1% of patients who initially appeared to present with CLBP were subsequently excluded during the consultation after imaging findings suggested serious spinal pathology. Whilst our study does not determine how such cases would be identified in the absence of imaging, the presence of serious pathology in a small proportion underscores the importance of thorough diagnostic evaluation, given the potential impact of missed cases at scale.

The imaging-based experiment in this study was exploratory and not intended to reflect real-world surgical decision-making. Agreement between patients’ reports of being offered lumbar spine surgery during consultation and decisions to offer surgery based on MRI reports alone was observed in only one out of three patients. These findings provide support for current guidance recommending that MRI findings alone should not drive surgical decision-making. In addition, surgeons categorised two thirds of the scans as indicative of nerve pain involvement in the leg, although all patients reporting leg pain had been excluded from the sample. Our findings suggest that patients’ preferences, regardless of other factors, may influence patients’ reports of being offered lumbar spine surgery during consultations.

The study examined factors associated with patients’ reports of being offered lumbar spine surgery among people with CLBP. This contributes to an area of the literature that is under-researched and is, to our knowledge, the first of its kind in the UK. Of importance, predictive factors were measured prior to the first contact with surgeons, so could not have been influenced by their explanations. The study was conducted in several hospitals, with a variety of surgeons and a reasonable sample size of patients.

However, several limitations are important to consider when interpreting our findings. The study exclusively focused on participants with CLBP, which may limit the generalisability of the findings to people with multiple comorbid chronic pain conditions. Although this was a multisite study, hospitals may not be representative of wider national or international practice. Furthermore, as MRI was performed prior to consultation in the included analytic sample, excluding patients without pre-consultation imaging may limit the generalisability of findings to settings where imaging is not routinely performed before specialist consultation.

Additionally, factors that may influence decision-making, such as ethnicity and the deprivation index, were not examined and should be explored in future research. We used a single item to measure surgical expectations. While assumptions of linearity were not violated, future research should consider using a validated, multi-item measure to explore this further. Moreover, the number of previous consultations was self-reported and may be prone to recall bias. Notably, this study did not explicitly ask surgeons for their primary reasons for offering lumbar spine surgery. It may have overlooked additional factors that may be relevant to their decision to offer surgery, particularly findings from physical examinations. In addition, patients’ reports of whether lumbar spine surgery was recommended may not perfectly reflect surgeons’ intended recommendations, as patients may interpret discussions about possible procedures as an offer of surgery. Future research is needed to explore surgeons’ decision-making processes to determine whether patients offered lumbar spine surgery have reasonable underlying causes amenable to it and which other factors have influenced the decision to offer surgery. Surgical approaches to CLBP vary significantly among surgeons within and across countries; therefore our findings may be unique to the UK health system. Finally, we did not follow-up patients who reported being offered lumbar spine surgery to determine who elected to undergo surgery and how this impacted their pain outcomes. Although the sample size met recommended criteria for logistic regression, smaller associations may have remained undetected.

According to the NICE guidelines recommend imaging in specialist settings such as those in this study only when it is expected to inform management. In our study, all patients had imaging, either prior to, during, or as a requirement for specialist consultation, suggesting that MRI scans are considered an essential part of decision-making pathways in these settings. Best practice recommends MSK specialists (e.g. physiotherapists, GPs with special interest) assess patients first, limiting imaging to clinically necessary cases. Future research should explore triaging pathways and referral processes in specialist settings. Patients who strongly expected to be offered lumbar spine surgery and had exhausted other interventions were more likely to report being offered surgery, even though current evidence suggests limited benefit and potential risks for many individuals with CLBP. This underscores the complexity of decision-making in this context, and future research is needed to understand how such factors shape real-world decision-making.
